# Case report: Onyx embolization of tentorial dural arteriovenous fistula *via* the meningohypophyseal trunk and medial tentorial artery of Bernasconi-Cassinari

**DOI:** 10.3389/fneur.2022.904877

**Published:** 2022-08-10

**Authors:** Kun Hou, Jinlu Yu

**Affiliations:** Department of Neurosurgery, The First Hospital of Jilin University, Changchun, China

**Keywords:** tentorial dural arteriovenous fistula, meningohypophyseal trunk, medial tentorial artery of Bernasconi-Cassinari, Onyx, embolization

## Abstract

For tentorial dural arteriovenous fistula (TDAVF), the meningohypophyseal trunk (MHT), and medial tentorial artery (MTA) of Bernasconi-Cassinari are rarely used as transarterial paths to perform the successful endovascular treatment (EVT). We reported a TDAVF mainly fed by the MHT. Onyx-18 casting in the MTA of Bernasconi-Cassinari under the assistance of coil embolization in proximal MHT was performed. The technique was reported in case 1. At the same time, case 2 with a similar TDAVF was chosen as a control. In case 1, a 52-year-old man suffered a cerebellar hemorrhage. A TDAVF was confirmed by computed tomography angiography and digital subtraction angiography. The feeding arteries included the MHT, middle meningeal artery (MMA), and the artery of Wollschlaeger and Wollschlaeger of the superior cerebellar artery. The MHT and MTA of Bernasconi-Cassinari were hypertrophied. First, a Marathon microcatheter was placed in the MTA to wait for Onyx casting, and then an Echelon-10 microcatheter was placed in the proximal MHT trunk with an aneurysmal dilation to perform coiling to prevent Onyx reflux. Then, Onyx casting obliterated the TDAVF. Case 2 was a 75-year-old woman with TDAVF, and the MTA of Bernasconi-Cassinari was the main feeder. First, the TDAVF experienced incomplete EVT with Onyx casting *via* the MTA under no assistance of coil embolization in the proximal MTA. The second EVT had to be performed *via* MMA. Then, Onyx casting obliterated the TDAVF. Therefore, for selected TDAVFs with hypertrophied MHT, under the assistance of coil embolization in proximal MHT, Onyx casting *via* MHT can finish the complete EVT.

## Introduction

Among all intracranial dural arteriovenous fistulas (DAVFs), tentorial DAVF (TDAVF) has a 4–8% prevalence (1). Due to high hemorrhagic risk, TDAVF requires appropriate treatments (2). Currently, endovascular treatment (EVT) with a liquid embolic agent is a valid, effective, and safe alternative for achieving complete occlusion in the majority of TDAVFs, in which the transarterial approach is still a first-line strategy, although the transvenous approach may be attempted (3, 4). However, EVT of TDAVF remains challenging (5).

Feeders of dural arterial origin of TDAVFs can include the middle meningeal artery (MMA), meningohypophyseal trunk (MHT), and medial tentorial artery (MTA) of Bernasconi-Cassinari, a meningeal branch of the occipital artery, a posterior meningeal artery from the vertebral artery, etc. (1). In addition, the meningeal branches of intracranial pial arteries can be involved as feeders, such as the artery of Davidoff and Schecter of the posterior cerebral artery, the artery of Wollschlaeger and Wollschlaeger of the superior cerebellar artery (SCA) (6, 7).

In theory, all feeding arteries can be used as transarterial paths, in which the MMA is commonly used to finish the complete obliteration of the TDAVF (8). However, when MMA is hypoplastic and too slim to act as a transarterial path, the MHT and MTA of Bernasconi-Cassinari can rarely be used as a transarterial path (3, 4). Here, we report a TDAVF with a dilated MHT as a feeder, casting Onyx-18 in the MTA of Bernasconi-Cassinari under the assistance of coil embolization in the proximal MHT with an aneurysmal dilation that finished the EVT. The technique was reported. At the same time, another similar TDAVF was chosen as a control.

## Case reports

### Case 1

**Figure 1:**
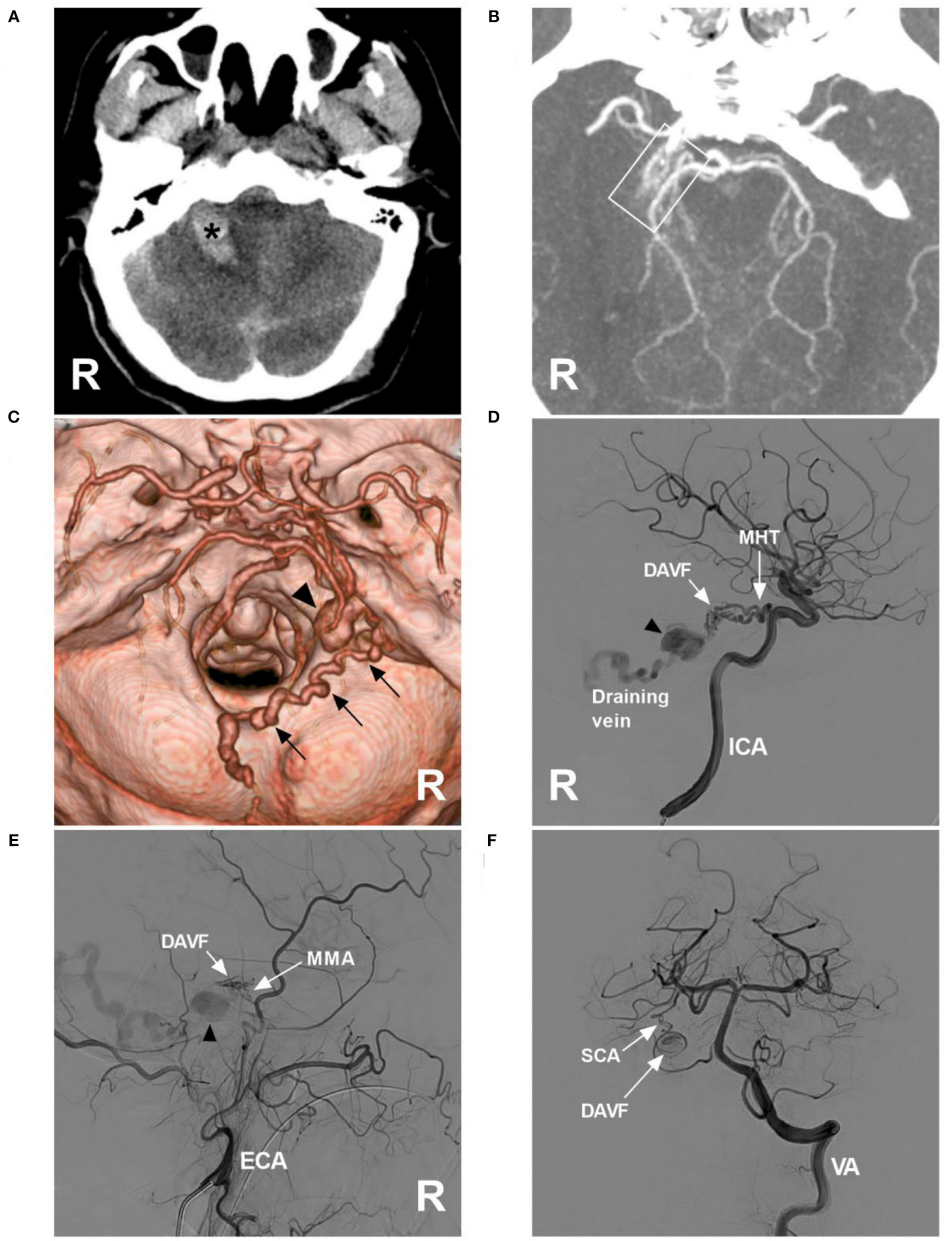
Preoperative images in case 1. **(A)** CT showing right cerebellar hemorrhage (asterisk). **(B)** Maximum intensity projection of CTA showing a tangle of abnormal vessels (frame) at the right margin of the tentorium; a DAVF was suspected. **(C)** Three-dimensional reconstruction of CTA showing a tortuous draining vein (multiple arrows) along the cerebellopontine fissure, the surface of inferior cerebellar hemisphere, and inferior vermin, went into the torcular herophili, with varix (triangle) at the beginning. **(D)** DSA of the right ICA showing that the MHT was the main feeding artery, and there was varix on the draining vein (triangle). **(E)** DSA of the right ECA showing that the petrous branch of the MMA was a minor feeding artery, and varix (triangle) was indicated. **(F)** DSA of the VA showing that the artery of Wollschlaeger and Wollschlaeger of the SCA was the minor feeding artery. CT, computed tomography; CTA, CT angiography, DAVF, dural arteriovenous fistula; DSA, digital subtraction angiography; ECA, external carotid artery; ICA, internal carotid artery; MHT, meningohypophyseal trunk; MMA, middle meningeal artery; R, right; SCA, superior cerebellar artery; VA, vertebral artery.

A 52-year-old man with an unremarkable medical history presented with acute onset of headache and then became drowsy. He was a patient of the Mongol nationality who lived in China, was healthy, and denied having a history of chronic diseases. He had no history of drug abuse or surgical treatment of craniocerebral disease. On physical examination, the patient was weakened and could not answer the questions correctly. His limbs had grade V muscle strength. His neck was stiff. The Babinski sign was positive in both lower limbs. CT showed cerebellar hematoma, subarachnoid hemorrhage (SAH), and hydrocephalus (Figure 1A). CT angiography (CTA) indicated a suspected DAVF of the tentorial margin, and a tortuous draining vein with varix was shown clearly, draining to the torcular herophili (Figures 1B,C). Digital subtraction angiography (DSA) confirmed the TDAVF and that the feeding arteries included the MHT, MMA, and artery of Wollschlaeger and Wollschlaeger of the SCA, in which the MHT was the main feeder (Figures 1D–F). The DAVF was Cognard Type IV and ruptured.

**Figure 2:**
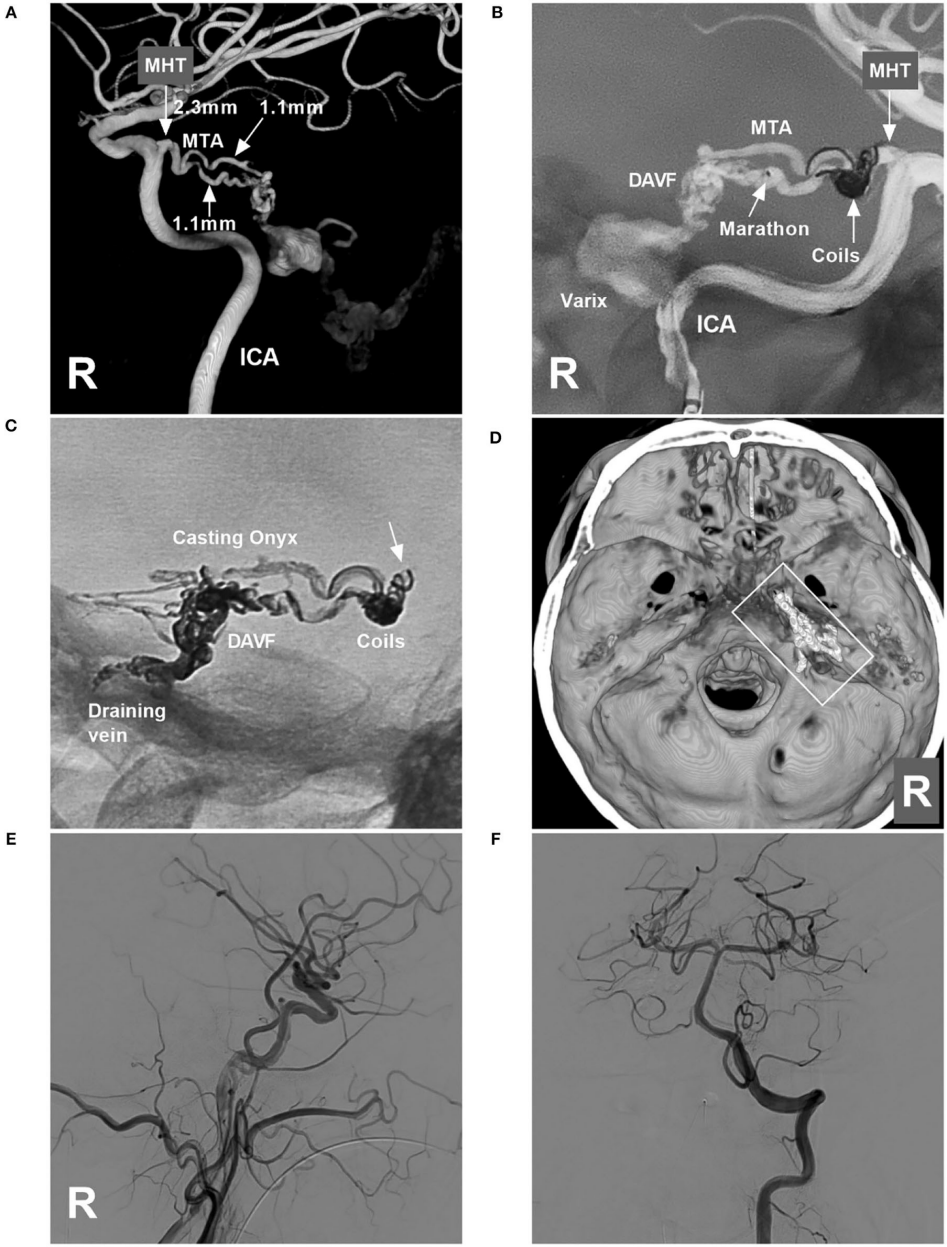
Operative images in case 1. **(A)** Three-dimensional reconstruction of DSA of the right ICA showing the diameters of the feeding artery to the TDAVF. The MHT origin was 2.3 mm, and the MTA of Bernasconi-Cassinari was 1.1 mm in diameter. **(B)** Road map of the ICA showing a Marathon microcatheter placed in the lower MTA. The MHT trunk with an aneurysmal dilation was coiled to produce the mass effect. **(C)** X-ray film showing the coils and casting Onyx. Onyx reflux reached the coils (arrow) but did not extend beyond the coils. **(D)** Three-dimensional reconstruction of Xper-CT showing the location of the Onyx casting (frame) at the margin of the tentorium. **(E,F)** DSAs of the right carotid artery **(E)** and VA **(F)** show complete obliteration of the DAVF. DAVF, dural arteriovenous fistula; DSA, digital subtraction angiography; ICA, internal carotid artery; MHT, meningohypophyseal trunk; R, right; MTA, medial tentorial artery; VA, vertebral artery.

Onyx-18 (Medtronic, Irvine, CA, USA) embolization *via* MHT was planned. Three-dimensional reconstruction of the internal carotid artery (ICA) showed the best projection degree of the MHT and MTA of Bernasconi-Cassinari (Figure 2A). First, a Marathon microcatheter (Medtronic, Irvine, CA, USA) was placed in the MTA, and then an Echelon-10 microcatheter (Medtronic, Irvine, CA, USA) was placed in the MHT. The proximal MHT trunk with an aneurysmal dilation was embolized with coils [Axium Prime 3.5 mm × 10 cm, 2 mm × 8 cm (Medtronic, Irvine, CA, USA)] to produce a mass effect to prevent Onyx reflux (Figure 2B). Then, the Onyx casting penetrated the fistula into the draining vein (Figures 2C,D). DSA of the carotid artery and vertebral artery confirmed that the TDAVF was completely obliterated (Figures 2E,F). Postoperatively, external ventricular drainage was performed, and the patient recovered gradually. One and a half months later, the patient could walk and answer the questions correctly.

### Case 2

**Figure 3:**
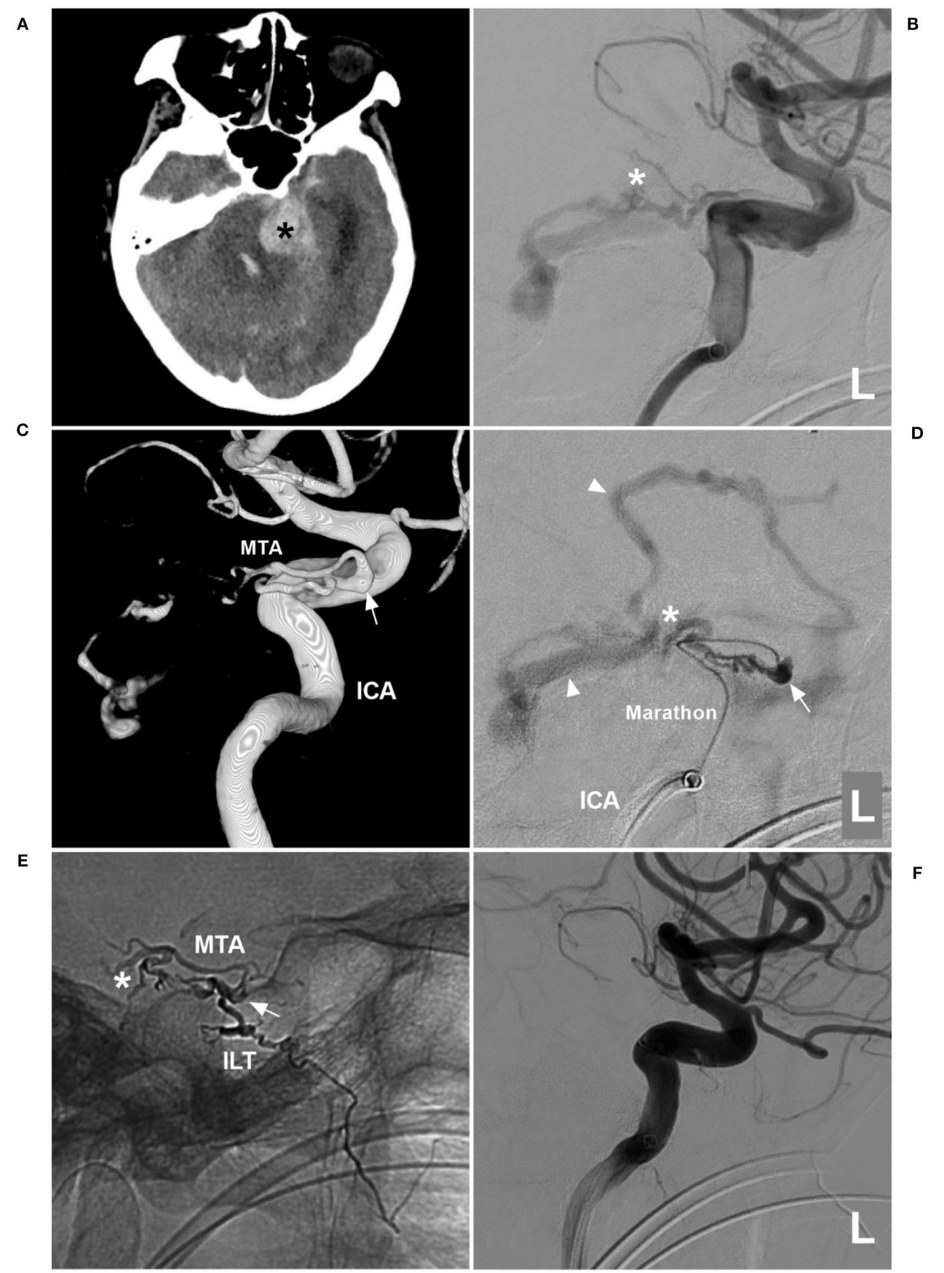
First EVT *via* MTA in case 2. **(A)** CT showing left cerebellar hemorrhage (asterisk). **(B)** DSA of the left ICA showing that the MTA of Bernasconi-Cassinari was the feeding artery to a DAVF (asterisk). **(C)** Three-dimensional DSA showing that double MTAs should share the common trunk with the ILT of the cavernous ICA; the arrow indicates the origin of these vessels. **(D)** Superselective angiogram *via* a Marathon microcatheter showing the TDAVF angioarchitecture. The arrow indicates the dilated beginning of the MTA, the asterisk indicates the fistula point, and the triangles indicate draining veins. **(E)** X-ray film showing Onyx casting. The arrow indicates the beginning of the MTA, the asterisk indicates that Onyx casting reached the fistula point *via* the MTA, and the Onyx also went into the ILT. **(F)** DSA of the left ICA showing that the DAVF was not seen. CT, computed tomography; DAVF, dural arteriovenous fistula; DSA, digital subtraction angiography; EVT, endovascular treatment; ICA, internal carotid artery; ILT, inferior lateral trunk; L, left; MTA, medial tentorial artery.

A 75-year-old woman with a history of hypertension presented with acute onset of headache and then fell into a coma. She was a patient of the Mongol nationality who lived in China. She had no history of drug abuse or surgical treatment of craniocerebral diseases. On physical examination. She was in a coma. Her limbs had grade II muscle strength. The Babinski sign was positive in both lower limbs. CT showed cerebellar hematoma, intraventricular hemorrhage, and hydrocephalus (Figure 3A). DSA confirmed the TDAVF and showed that double MTAs of Bernasconi-Cassinari were the main feeding arteries. Double MTAs shared the common trunk with the inferior lateral trunk (ILT) of the cavernous ICA (Figures 3B,C). The DAVF was Cognard Type IV and ruptured.

**Figure 4:**
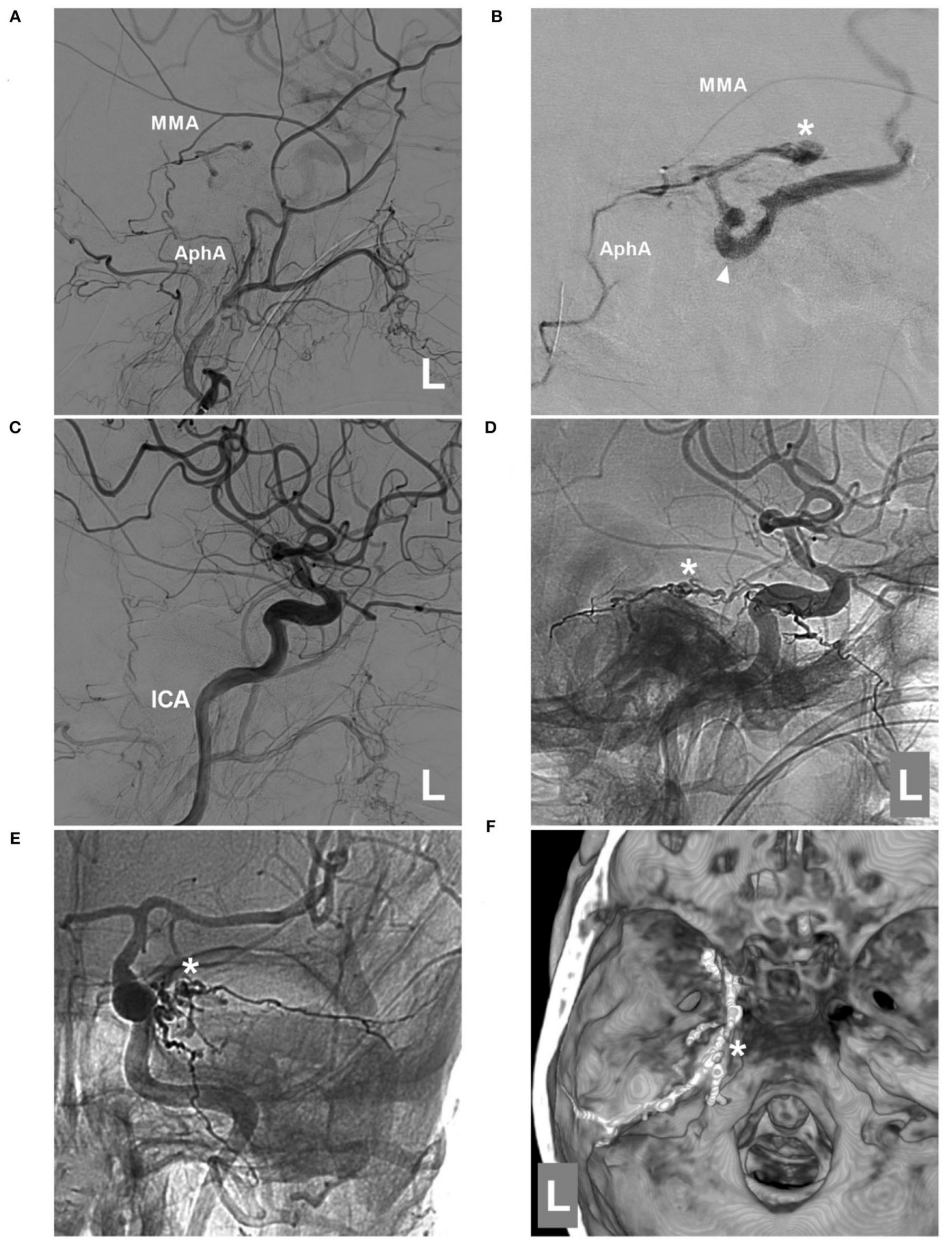
Second EVT *via* MMA in case 2. **(A)** DSA of the left external carotid artery showing that the MMA and AphA still supplied the DAVF. **(B)** Superselective angiogram of the MMA *via* a Marathon microcatheter showing the DAVF angioarchitecture. The asterisk indicates the fistula point, and the triangle indicates the draining vein. **(C)** DSA of the left carotid artery showing that the DAVF was completely embolized after Onyx casting *via* the MMA. D-E: Lateral view **(D)** and anteroposterior **(E)** unsubtracted DSA showing the casting Onyx; the asterisk indicates the fistula point. **(F)** Three-dimensional reconstruction of Xper-CT showing Onyx casting. The asterisk indicates the fistula point at the margin of the tentorium. AphA, ascending pharyngeal artery; DAVF, dural arteriovenous fistula; DSA, digital subtraction angiography; EVT, endovascular treatment; ICA, internal carotid artery; L, left; MMA, middle meningeal artery.

EVT was planned *via* the MTA of Bernasconi-Cassinari. First, a Marathon microcatheter tried to go into the distal MTA, but it could only be placed at the tortuous MTA beginning (Figure 3D). Then, Onyx-18 was cast (Figures 3E,F). EVT was incomplete, and DSA of the external carotid artery showed that the MMA and ascending pharyngeal artery still supplied the TDAVF (Figures 4A,B). The second Onyx casting was performed by a Marathon microcatheter *via* the MMA, and the TDAVF was cured (Figures 4C–F). Postoperatively, external ventricular drainage was performed, and she did not recover gradually. She was still in a coma 1 month later.

## Discussion

Compared with DAVFs in other locations, 60–75% of TDAVFs are prone to hemorrhage due to venous hypertension caused by retrograde leptomeningeal drainage, including the supra- and infratentorial veins, as well as the deep veins of the brain stem (Cognard Type III or IV) (9–11). Thus, aggressive intervention is necessary (10). In a systematic review by Cannizzaro et al., over the past 35 years, TDAVFs have increasingly been treated with EVT, and the use of the Onyx liquid embolic system may continue to shift the balance increasingly toward EVT (12). Of all feeding arteries that can potentially be used as transarterial paths to perform EVT, the MMA is still the gold transarterial path to complete the obliteration of the TDAVF (7). The MHA and MTA of Bernasconi-Cassinari are challenging for TDAVF, and the success rate is low, such as in case 2.

Normally, the MHT originates in the posterior genu of the cavernous ICA, with a diameter of approximately 1 mm, and then trifurcates into the MTA of Bernasconi-Cassinari, dorsal meningeal artery (the dorsal or lateral clival artery), and inferior hypophyseal artery (13–15). Ninety percent of MTAs are usually the terminal branch of the MHT, and 10% of them can arise directly from the cavernous ICA; 80% of MTAs originate as a single branch and as a bifurcation and trifurcation in 20% (16). In this report, case 1 had an MTA that arose from MHT, case 2 had an MTA that directly arose from the cavernous ICA, and the MTA in two cases was the bifurcation pattern. The MTA travels along the tentorium just lateral to the tentorial incisure and contributes to the supply of the medial portion of the tentorium. A normal MTA is slim, and its diameter was 0.7 mm in the Banerjee et al. specimen (16) and 0.5 in the Peltier et al. specimen (17). The MTA had a length of 15 mm in the Banerjee et al. specimen (16) and 21.7 mm in the Peltier et al. specimen (17).

According to these normal anatomical parameters, the MHT and MTA of Bernasconi-Cassinari were not appropriate for use as the transarterial path of the current EVT. Under the increased blood flow in TDAVFs, the MHT and MTA can be hypertrophied to provide accessible catheterization. In case 1, the MHT reached 2.3 mm, and the MTA reached 1.1 mm in diameter. However, catheterization of the MHT and MTA of Bernasconi-Cassinari is still difficult due to tortuosity (18). In addition, the allowable reflux distance of Onyx was too short for them.

Therefore, EVT with the MHT and MTA of Bernasconi-Cassinari as the transarterial path is rarely performed. In Rezende et al.'s report with a large series of 45 TDAVFs treated with transarterial embolization, the MTA path was used only once (2.2%, 1/45) (4). Van Rooij et al. studied the feasibility of the MTA as a transarterial path to embolize TDAVFs, with a balloon temporarily deployed within the ICA in front of the MHT origin to stabilize the microcatheter and prevent reflux of embolic material into the ICA (19). However, the technique is still difficult to apply. Recently, the Scepter Mini dual-lumen balloon microcatheter (MicroVention, Aliso Viejo, CA, USA) with a 1.6 French distal profile and 2.2 mm balloon at its tip were introduced (20). It can avoid the Onyx reflex in embolizing DAVFs (21). However, the Scepter Mini balloon microcatheter is stiff and thick for the catheterization for MHT and MTA of Bernasconi-Cassinari.

In case 1, the MHT and MTA were hypertrophied, and the current softest and thinnest Marathon microcatheter had accessed the fistula. Therefore, during Onyx casting, if too much reflux can be prevented, the TDAVF can be cured. Due to an aneurysmal dilation at the proximal MHT, we planned to coil the aneurysmal dilation to produce a mass effect to prevent Onyx reflux. The technique was similar to that reported by Chapot et al.; it was designed to create a plug by trapping the Onyx-compatible microcatheter with coils to obtain wedge-flow conditions (22). With the assistance of the technique, the TDAVF was cured in case 1. Therefore, the technique is feasible only for highly selective TDAVFs. As a control of case 2, the MTA of Bernasconi-Cassinari was dilated, but the MTA was tortuous, the microcatheter did not access the fistula point, and without coiling to prevent Onyx reflux, complete EVT was not achieved.

In our report, we chose Onyx-18 as an embolic agent because Onyx-34 with a high viscosity was not appropriate (23). In case 1, the Onyx reflux reached the coils, but the reflex did not penetrate them. Therefore, this technique cannot typically be used because of the danger associated with possible reflux into the ICA, which should not be underestimated. Prior to this technique, all other paths should be exhausted, especially the MMA path, which has been attempted (24). In addition, microsurgery should be considered as a surgical approach, especially for TDAVF with a single draining vein into the posterior fossa (25).

In case 1, after finishing Onyx embolization, the Marathon microcatheter can be easily removed without difficulty, which was suggested in our previous reports (26, 27). When removing the Marathon microcatheter, backing mass coils toward the ICA should be considered. However, in case 1, the proximal MHT had an aneurysmal dilation, and the coils were stable. In addition, the Echelon-10 microcatheter should not be removed too early; it can support coils when removing the Marathon microcatheter. In addition to the Marathon microcatheter, the Apollo detachable tip microcatheter (Medtronic, Irvine, CA, USA) was an alternative for case 1 (28). However, the detachable tip lengths of 15 and 30 mm were too long, and the detachment zone may be located in the ICA. After detachment, the detachable tip in the ICA was detrimental.

## Conclusion

Currently, for TDAVFs, if there is no blood supply from the MMA, the MHT and MTA of Bernasconi-Cassinari are highly hypertrophied. With the assistance of coil embolization in the proximal MHT, Onyx casting *via* the MHT and MTA can finish the complete EVT.

## Limitation

This is a report, and the conclusion should be cautiously interpreted. In addition, no case of surgical treatment can be provided as a comparison with EVT, which was a limitation.

## Data availability statement

The original contributions presented in the study are included in the article/supplementary material, further inquiries can be directed to the corresponding author.

## Ethics statement

Ethical review and approval were not required for the study on human participants in accordance with the local legislation and institutional requirements. Written informed consent was obtained from the individual(s) for the publication of any potentially identifiable images or data included in this article.

## Author contributions

JY designed the study and drafted the manuscript. KH collected the data. JY and KH confirm the authenticity of all the raw data. Both authors have read and approved the final manuscript.

## Conflict of interest

The authors declare that the research was conducted in the absence of any commercial or financial relationships that could be construed as a potential conflict of interest.

## Publisher's note

All claims expressed in this article are solely those of the authors and do not necessarily represent those of their affiliated organizations, or those of the publisher, the editors and the reviewers. Any product that may be evaluated in this article, or claim that may be made by its manufacturer, is not guaranteed or endorsed by the publisher.
